# Research on the Privacy Security of Face Recognition Technology

**DOI:** 10.1155/2022/7882294

**Published:** 2022-01-25

**Authors:** Luning Pang

**Affiliations:** School of Law, The University of Melbourne, Melbourne 3002, Australia

## Abstract

To solve the problem that privacy data are easy to leak in the application of face recognition technology in apps, a method which is based on differential privacy for privacy security protection is proposed. Firstly, Bayesian GAN is conducted to obtain the training data with the same distribution as the privacy data, and the algorithm of differential privacy is conducted to train the training data to obtain these labels with privacy protection. Then, based on the proposed lightface lightweight face recognition model, the tag with noise is generated, and the gradient descent is conducted on the recovered face feature vector from the attack. Finally, through the analysis of privacy loss, an accurate privacy protection boundary is provided. From the results of experiments, it could be known that the proposed privacy security protection method can effectively protect the parameter information of the face recognition model under the face recognition technology and reduce the recognition accuracy of the image recovered by the attacker. Compared with the privacy protection methods such as DPSGD and PATE, it has strong privacy protection ability and can be applied to the privacy protection of practical APP.

## 1. Introduction

Since the new century, the application of the Internet industry and the rapid development of computer technology have not only brought great convenience to data sharing but also increased the risk of privacy leakage. In recent years, with the enrichment of network attack means and the frequent occurrence of security events such as privacy leakage, the protection of privacy data is no longer simply hiding sensitive attributes in data but more to maximize the accuracy of data query and minimize the possibility of identification and recording, which poses a new challenge to privacy protection, especially today when face recognition technology is becoming more and more mature.

In addition, the researchers also did a lot of research on face recognition and privacy protection. First of all, in the field of face recognition, Shoba et al. put forward that the accuracy of face recognition can be improved through efficient feature extraction [1]. Singha et al. fused AOS and VGG to extract and fuse the face features and then recognized faces. The characteristic of this method is that the accuracy of recognition can be greatly improved through deep fusion [2]. Sang et al. proposed an image noise removal method based on the hybrid norm constrained regression model for the noise problem in face recognition images. Finally, experiments are carried out in five commonly used face recognition databases. It could be known from the results that the abovementioned method is superior to the traditional regression model [3]. Padmanabhan et al. applied the machine learning algorithm to face recognition and verified the efficiency of machine learning algorithm in face recognition [4–8]. Liu et al. came up with a method of face image recognition which is based on singular value processing, so as to broaden the data sample set [9]. It can be seen from the abovementioned research that the current technology for face recognition mainly starts from three aspects: one is the feature extraction of the face image; the other is the image features classification; and the third is the image sample processing. However, the abovementioned research has one thing in common, that is, to improve the accuracy of face recognition.

To solve the problem of privacy security, Kumar et al. proposed to introduce biometric technology into cloud computing technology, such as face recognition technology, so as to improve the security of the cloud computing environment [10]. For the privacy protection of smart home system, Yassine et al. applied the recognition method of improved k-nearest neighbor classifier to it. The results show that the security is greatly improved by this method. However, generally , there are relatively few research studies on privacy protection of application programs [11].

Based on the large number of studies by the abovementioned scholars, deep learning has achieved good results in privacy protection under face recognition technology. Therefore, this study uses Bayesian generative adversarial networks (GANs) in deep learning to obtain the training data with the same distribution as the privacy data and proposes a privacy security protection method of differential privacy. The lightface lightweight face recognition model is constructed to generate labels with noise, the gradient descent is conducted on the recovered face feature vector from the attack, and the privacy loss analysis is used to obtain the accurate privacy protection boundary so as to realize the privacy protection under the face recognition technology.

## 2. Basic Method

### 2.1. Introduction to Bayesian GAN

The Bayesian GAN is a variant of the GAN. Bayesian formula is used to train the GAN to realize unsupervised and semisupervised learning, which can solve the problems of gradient disappearance and training instability in the training process of the GAN. In the Bayesian GAN, the weight vectors *θ*_*x*_ and *θ*_d_ of generator and recognizer can be obtained by iterative sampling of conditional posterior probability [12–14]:

(1) $$\begin{array}{c} p\left(\theta_{g}|z,\theta_{d}\right)\infty \left(\overset{n_{x}}{\underset{i=1}{\prod }}\sum_{y=1}^{K}D\left(G\left(z^{\left(i\right)};\theta_{x}\right)=y^{\prime },\theta_{d}\right)\right)p\left(\theta_{g}|a_{k}\right), \end{array}$$

(2) $$\begin{array}{c} p\left(\theta_{d}|z,X,Y_{k},\theta_{g}\right)\infty \overset{n_{d}}{\underset{I=1}{\prod }}\sum_{y=1}^{K}D\left(x^{\left(j\right)}=y^{\prime },\theta_{d}\right)\overset{n_{d}}{\underset{l=1}{\prod }}D\left(G\left(z^{\left(t\right)};\theta_{g}\right)\overset{N_{a}}{\underset{j=1}{\prod }}\left(D\left(x_{x}^{\left(y\right)}=y_{s}^{\left(y\right)};\theta_{cf}\right)\right)p\left(\theta_{d}|a_{d}\right)\right), \end{array}$$

where *K* is the amount of classes; *N*_*s*_ is the amount of samples which keep labels; *z* stands for white noise; *n*_*g*_ and *n*_*d*_ are, respectively, the amount of small-lot training samples for generators and recognizers; *a*_*g*_ and *a*_*d*_ are hyperparameters; *p*(*θ*_*g*_*|a*_*g*_) and p(*θ*_*d*_*|a*_*d*_) are the prior probabilities of generator and recognizer parameters under *a*_*g*_ and a_*d*_; *D*(*x*_*x*_^*t*^=*y*_*s*_^(*y*)^; *θ*_*d*_) is the probability that sample *x*_*s*_^(*y*)^*N* belongs to tag *y*_*s*_^(*i*)^; 0 means that the generator generates sample class labels.

Assuming that the input of the Bayesian GAN is *x*, the prediction distribution whose output is *y*_*∗*_ can be expressed as [15]

(3) $$\begin{array}{c} p\left(y_{*}|X_{*},D\right)=\int p\left(y_{*}|X_{*},\theta_{d}\right)p\left(\theta_{d}|D\right)d{\theta \;}_{d}\approx \frac{1}{T}\sum_{k=1}^{T}p\left(y_{*}|X_{*},\theta_{d}^{\left(k\right)}\right),\theta_{d}^{\left(k\right)}∼p\left(\theta_{d}|D\right). \end{array}$$.

In the Bayesian GAN, HMC of the dynamic gradient is conducted to make the weights of the generation network and discrimination network to proceed marginalization, and this method can get good performance without any standard intervention.

### 2.2. Differential Privacy Introduction

Differential privacy is a new technology for privacy protection whose purpose is to make the accuracy of data query to the maximum and the possibility of identification and recording to the minimum. The basic conceptual requirement is that each single element in the dataset has limited impact on the overall output of the dataset. Therefore, this technology can ensure that after the attacker queries the output results of the dataset, the attacker still cannot infer which element in the dataset has the impact on the output results and then cannot infer the relevant individual privacy information in the dataset to realize privacy protection.

The definition of the form of differential privacy is represented as follows:

**Definition 1 .**

Let the values of all output results of the random algorithm *m* be PM and SM be a subset of PM, then for any pair of *D* and *D*′ which are adjacent datasets, *m* satisfies *ε* difference privacy, as shown in equation (4) [16]:

(4) $$\begin{array}{c} P_{r}\left[M\left(D\right)\in S_{m}\right]\leq e^{\varepsilon }\mathrm{Pr}\left[M\left(D^{\prime }\right)\in S_{m}\right], \end{array}$$

where the parameter ∈ stands for the budget of privacy protection, and the smaller the value of *ε*, the higher the level of privacy protection.

According to Definition 1, differential privacy limits the influence of any element in the dataset on the output result of the algorithm and theoretically ensures that the algorithm meets *ε* differential privacy, which can be realized by adding noise in practical application.

### 2.3. Introduction to Lightface Model

The lightface model is the first lightweight network model based on the deep decomposable convolution model MobileNets by adding a weight calibration module after the convolution layer. The first layer is a standard convolution layer, and the last layer is a full-connection layer, and the other network layers are the relu activation function and batch normalization. The weight calibration module is located behind the convolution layer, and its concrete structure is shown in Table 1 [17].

In Table 1, *D*_*F*_ × *D*_*F*_ stands for the size of the feature map which is input, and *M* stands for the amount of feature map channels. FC1 and FC2 are full-connection layers, similar to the BP neural network. In FC1, the dimension is reduced by adding the *γ* parameter to reduce the amount of parameters. In FC2, appropriate *γ* is set to raise the dimension to the level before dimension reduction. In this paper, let *γ* be 8.

Through the weight calibration module, the weight of each channel can be obtained, and then a new feature map can be obtained by learning the weight and weighting each channel. Finally, the obtained feature map is reduced to 1 × 1 dimension through the last average pool layer of the lightface model, and the results are output through the full-connection layer, and at the same time, L2 feature normalization is carried out to obtain the feature representation. If the L2 norm value is set to 1, all features of the image would be mapped to a hypersphere. This feature is used to calculate the triple loss, and the feature is optimized according to the loss result and whether the two images are of the same class can be judged by the point distance in the feature space.

The triplet loss is calculated as follows [18]:

(5) $$\begin{array}{c} {\left\|f\left(x_{i}^{A}\right)-f\left(x_{x_{i}}^{p}\right)\right\|}_{2}^{2}+a<{\left\|f\left(x_{i}^{l}\right)-f\left(x_{i}^{N}\right)\right\|}_{2}^{2}\forall \left(f\left(x_{i}^{A}\right),f\left(x_{i}^{p}\right),f\left(x_{i}^{N}\right)\right)\in T. \end{array}$$

In the formula, *f*(*x*_*i*_^*A*^), *f*(*x*_*i*_^*p*^), *f*(*x*_*i*_^*N*^) represent the characteristic expression of samples *A*, *p*, and *N*, respectively, and *p* and *N* are positive samples which are the same kind as *A* and negative samples which are the different kind as *A*, respectively. *T* represents the possible triple combination of the training set, and the amount of elements of *T* is *M*. Therefore, the corresponding objective function can be represented as

(6) $$\begin{array}{c} {\sum_{i}^{M}\left[{\left\|f\left(x_{{}_{t}}^{A}\right)-f\left(x_{i}^{P}\right)\right\|}_{2}^{2}-{\left\|f\left(x_{i}^{A}\right)-f\left(x_{i}^{N}\right)\right\|}_{2}^{2}+a\right]}_{+}. \end{array}$$

According to the objective function, when the distance between *A* and *N* is greater than or equal to the sum of *a* and the distance between *A* and *p*, the loss is 0; on the contrary, there will be losses.

According to the above analysis, the lightface model uses less multiplication and addition operations when defining the network, so it can enhance the computing speed of the network to a certain extent. However, this method has limited ability to improve the network computing speed, and for the sake of further improving the network computing speed, this paper uses the highly optimized general matrix multiplication GEMM function for convolution operation. Because the main amount of computation in the lightface is focused on 1 × 1 point convolution and the GEMM can directly realize 1 × 1 convolution operation, the GEMM can realize high-efficiency operation in lightface.

## 3. Face Recognition Method Based on Differential Privacy

### 3.1. Differential Privacy Framework

**Figure 1:**
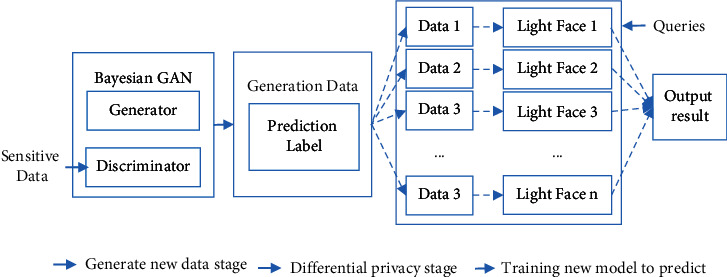
Privacy protection method under face recognition technology.

On the basis of the abovementioned basic knowledge, in this study, the privacy protection method under face recognition technology is designed as Figure 1. In the learning strategy stage, firstly, the Bayesian GAN is used to train the labeled private data, and the trained model is used to generate the new data. Then, the newly generated data will be input into the trained Bayesian GAN discriminator to obtain the prediction probability of each data. Finally, the generated data and its corresponding tags will be input into the data processing stage. By these steps, the data learning is completed.

In the privacy protection stage, the main purpose is to add uncertainty to the generated data tags to protect privacy, and this study is realized by adding noise satisfying the Laplace distribution. Firstly, suppose the dimension of each data label is *K* + *l*, the data of last one dimension represent the probability value of false data, and the probability that the data belongs to class *i* data is *P*_*i*_. Adding the noise shown in equation (7) to *P*_*i*_ and performing the normalization process shown in equation (8), a noisy label is generated [19].

(7) $$\begin{array}{c} P_{i}^{\prime }=P_{i}+Lap\left(\gamma \right), \end{array}$$

(8) $$\begin{array}{c} P_{i}^{n}=\frac{P_{i}^{\prime }}{\sum_{i=1}^{k}P_{i}^{\prime }}, \end{array}$$

where *γ* is the privacy protection parameter, indicating the strength of the privacy protection that can be provided, and the greater its value, the stronger the privacy protection provided. Lap(*γ*) represents the Laplace distribution with position 0 and scale *γ*. *P*_*i*_′ represents the added noise probability value. *P*_*i*_′ is the normalization result of *P*_*i*_′.

Finally, the data with noise labels are used to train the final release model. In order to enhance the accuracy of this model, the study uses the ensemble learning method for training the external access model. Firstly, *n* data are collected from the data for training the lightface model, and *n* lightface models are obtained. Then, the *n* data lightface models are aggregated into an output model. Finally, the final result of the input data can be obtained according to the majority principle, as follows:

(9) $$\begin{array}{c} f\left(\bar{x}\right)=\underset{j}{\arg\max}\left\{n_{j}\left(x\right)\right\}, \end{array}$$

where $f\left(\vec{x}\right)$ stands for the final output result of this model, $\vec{x}$ stands for the input data, and $n_{j}\left(\vec{x}\right)$ stands for the amount of results of $\vec{x}$ belonging to class *j*, *j* ∈ [*K*].

In the whole process of privacy protection, because the privacy data is just conducted for Bayesian GAN training and is not conducted in publishing model training, even if the external model parameters are disclosed, the attacker cannot get the privacy data by reconstruction attack. In addition, because the input data of the publishing model is the newly generated nonsensitive data of the Bayesian GAN and noise is added in the privacy protection stage, it will disrupt the attacker's reconstruction attack so that the attack cannot reproduce the clear privacy data. Finally, the Ensemble learning is adopted to train release model which ensures that the model has high accuracy while realizing privacy protection.

### 3.2. The Loss of Privacy Analysis

Based on the above differential privacy protection method, the privacy protection under face recognition technology can be ensured. In order to further accurately realize privacy protection, this study uses time accounting to analyze the loss of privacy in detail and provides an accurate privacy protection boundary.

#### 3.2.1. Time Accounting

Time accounting is a method to accurately calculate the loss of privacy and its basic definition is as follows:

**Definition 2 .**

Suppose there is an auxiliary input aux, and for output *o* ∈ *R*, its privacy loss is

(10) $$\begin{array}{c} c\left(o;M\left(aux\right)d,d\right)\overset{\Delta }{=}\log\frac{\mathrm{Pr}\left[M\left(aux,d\right)=o\right]}{\left[M\left(aux,d^{\prime }\right)=o\right]}, \end{array}$$

where *c*(*M*, *aux*, *d*, *d*′) represents the privacy loss random variable which is defined as *c*(*M*(*d*), *M*, *aux*, *d*, *d*′).

**Definition 3 .**

Suppose the auxiliary input of the random algorithm *M* : *D*⟶*R* is aux and *d* ⊕ *d*′ is the adjacent datasets, time accounting can be expressed as

(11) $$\begin{array}{c} a_{M}\left(\lambda \right)\overset{\Delta }{=}\underset{aux,d,d^{\prime }}{\max}a_{M}\left(\lambda ;aux,d,d^{\prime }\right), \end{array}$$

where *a*_*M*_(*λ*; *aux*, *d*, *d*′) represents the time generation function of privacy loss random variable, which can be calculated as follows:

(12) $$\begin{array}{c} a_{M}\left(\lambda ;aus,d,d^{\prime }\right)\overset{\Delta }{=}\;\log\;E\left[\exp\left(\lambda C\left(M,aux,d,d^{\prime }\right)\right)\right]. \end{array}$$

In addition, time accounting has the following properties.

**Theorem 1 (Composability).**

Assuming that *M* is the set of adaptive algorithms M1,..., MK, and *M*_′_ : ∏_*J*=1_^*i*−1^*R*_′_*∗D*⟶*R*_*l*_, then, any output sequence *O*_1_,…, *O*_*k*−1_, and *λ* satisfy [19]

(13) $$\begin{array}{c} a_{M}\left(\lambda ;d,d^{\prime }\right)=\sum_{i\Delta 1}^{k}a_{M}\left(\lambda ;o_{1},\ldots ,o_{\prime },d,d^{\prime }\right). \end{array}$$

**Theorem 2 .**

For any *ε* > 0, *m* satisfies the (*ε*, *δ*)− differential privacy:

(14) $$\begin{array}{c} \delta =\underset{\lambda }{\min}\exp\left(a_{M}\left(\lambda \right)-\lambda_{\varepsilon }\right). \end{array}$$

**Theorem 3 .**

Assuming that there are adjacent datasets *d*, *d*′, and *M* is an algorithm for recording *P*_*i*_+*Lap*(1/*γ*) that satisfies the (2*γ*, 0)− differential privacy, the corresponding number of votes NJ of each type can differ by 1 at most, and any *l*, aux, *d*, *d*′ satisfy [20–23]

(15) $$\begin{array}{c} a\left(l;aux,d,d^{\prime }\right)\leq 2\gamma^{2}l\left(l+1\right). \end{array}$$

#### 3.2.2. Privacy Analysis of Face Recognition Model with Privacy Protection

The privacy analysis of the face recognition model with privacy protection is conducted by time accounting. Firstly, in each step of model training, (2*γ*, 0)− differential privacy is selected to generate labels with privacy protection, and the algorithm meets $\left(4T\gamma^{2}+2\gamma \sqrt{2T\;\ln1/\delta }\delta \right)-$ differential privacy after *T* steps. Due to the wide range of privacy boundary, it is hard to satisfy the practical application demands of the face recognition model with privacy protection, so that Nicolas Papernot proposes a stricter privacy loss boundary method. This method satisfies the following theorem:

**Theorem 4 .**

If *M* satisfies (2*γ*, 0)− differential privacy, any output *o*^*∗*^ satisfies [24–26]:

(16) $$\begin{array}{c} q\geq \mathrm{Pr}\left[M\left(d\right)\neq o^{*}\right], \end{array}$$

(17) $$\begin{array}{c} l,\gamma \geq 0, \end{array}$$

(18) $$\begin{array}{c} q<\frac{e^{2\gamma }-1}{e^{4\gamma }-1}. \end{array}$$

Then, for any aux, *d*, *d*′, *M* satisfies

(19) $$\begin{array}{c} a\left(l;aux,d,d^{\prime }\right)\leq \;\log\left(\left(1-q\right){\left(\frac{1-q}{1-e^{2\gamma }q}\right)}^{l}+q\;\exp\left(2\gamma l\right)\right). \end{array}$$

**Theorem 5 .**

Suppose *n* is the label score vector of dataset *D* and *j* satisfies *n*_*j*^*∗*^_ ≥ *n*_*j*_, then,

(20) $$\begin{array}{c} \mathrm{Pr}\left[M\left(d\right)\neq j^{*}\right]\leq \sum_{i\neq j}\frac{2+\gamma \left(n_{j^{*}}-n_{j}\right)}{4\;\exp\left(\gamma \left(n_{i^{*}}-n_{i}\right)\right)}. \end{array}$$

According to the above properties, an upper limit *q* can be provided for a specific fractional vector *n* to realize the constraint on a specific time. Using *λ* to calculate a specific time, a more strict privacy boundary than time accounting will be obtained.

## 4. Results and Analysis

### 4.1. Experimental Environment and Data Source

In this experiment, under the framework of TensorFlow, the model will be trained. The experimental data are selected from LFW, YTF, and SFC datasets. Among them, the LFW dataset includes 13323 online pictures of 5749 people. The YTF dataset contains 3425 groups of YouTube videos with 1595 themes and in this experiment, 5 clear frames of images in each video are selected as experimental data. The SFC dataset includes 4.4 million images of marked faces of 4030 people, with 800–1200 data per person.

For the sake of making the images of the experimental dataset conform to the model input, the experimental images are preprocessed in this study. Firstly, the face detector is run on the experimental image to generate a tight bounding box around the face. Then, the generated face image is adjusted to the corresponding network input size and in this paper, its resolution is 224 ∗ 224. Finally, the image that meets the model input conditions can be input into the network for training.

### 4.2. The Parameter Setting with Bayesian GAN

**Figure 2:**
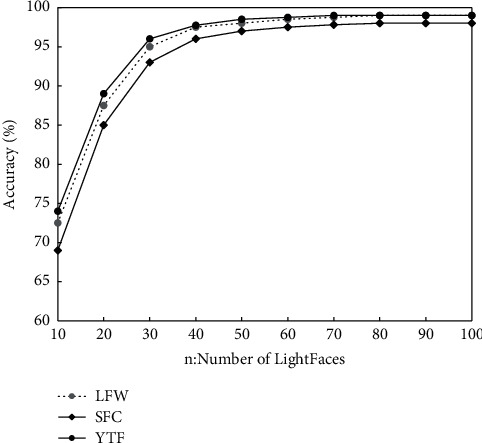
Accuracy of face recognition model under different number of lightfaces.

In this experiment, the initial learning rate of the lightface model is set to 0.05, the threshold a is 0.2, and the parameters is updated by using the asynchronous gradient descent algorithm. In order to determine the influence of the amount of lightfaces on the recognition accuracy of face recognition models, bootstrap sampling will be used to extract *n* data from LFW and YTF datasets, respectively, to train *n* models, and the results will be aggregated to obtain the accuracy of face recognition models under different lightface models, as represented in Figure 2. The figure shows that the accuracy of the face recognition model gradually increases with the increase of N, and the accuracy tends to be stable after *n* = 70. By fully considering the accuracy and size of the model, *n* = 50 is set in this study.

### 4.3. The Experimental Results

#### 4.3.1. Bayesian GAN Training Data Validation

**Figure 3:**
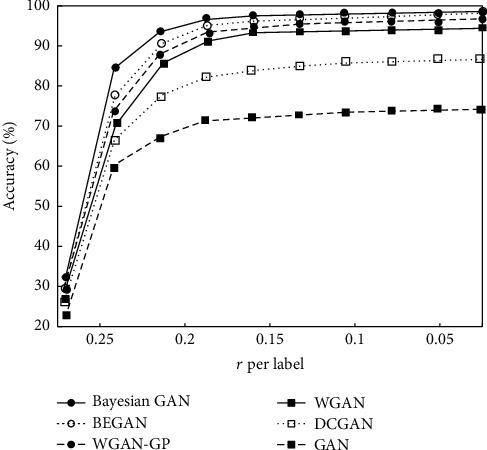
Recognition accuracy of the face recognition model of training samples generated by different GANs.

For the sake of verifying the effectiveness of the Bayesian GAN used in the paper, this experiment uses different GAN network models to generate the training data for face images with different noise levels under the TensorFlow framework and inputs the face recognition model, and the obtained accuracy results of the model are represented in Figure 3. The figure shows that the training data generated by the Bayesian GAN has the highest recognition accuracy of the face recognition model, and this advantage becomes more obvious with the enhancement of noise. This shows that the data generated by the Bayes GAN is more diverse and real and closer to the real sample distribution.

**Figure 4:**
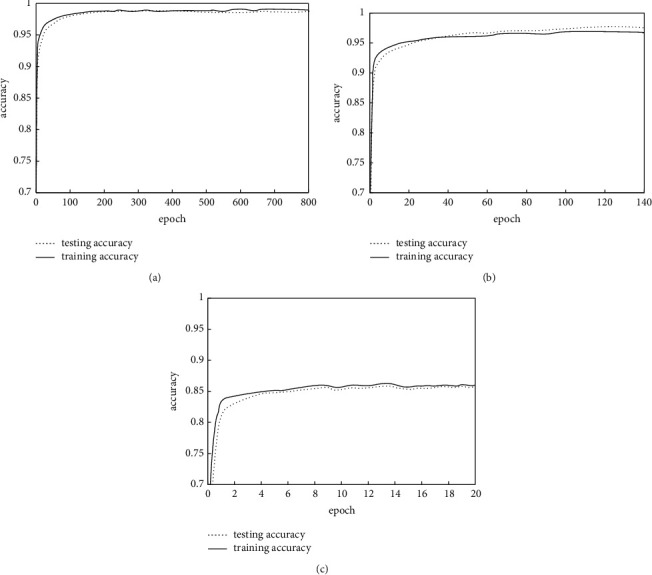
Accuracy of the face recognition model under different degrees of noise.

For the sake of further verifying the effectiveness of the Bayesian GAN, the paper adds noise to the data labels which are generated by the network, adds high noise with a value of 0.05, medium noise with a value of 0.1, and low noise with a value of 0.2, respectively; inputs the face recognition model for training; and obtains model training results with different noise levels, as shown in Figure 4. The figure shows that with the enhancement of noise intensity, the recognition accuracy of data labels and face models generated by the Bayesian GAN gradually decreases. Under the conditions of low noise, medium noise, and high noise, the accuracy of model recognition can reach 99%, 97%, and 86%, respectively. This shows that the Bayesian GAN has certain effectiveness, which can guarantee that the face recognition model has high recognition accuracy and meet the needs of practical application.

#### 4.3.2. Lightface Model Validation

*(1) Model Comparison*. For the sake of verifying the effectiveness of the lightface model, the parameters and calculation of this model and of other face recognition models have been compared, as represented in Table 2 [17]. The table shows that compared with other models, the amount of calculation and parameters of the lightface model proposed in this study are greatly reduced, indicating that the model proposed in this study has certain advantages, and the speed and volume meet the requirements of the lightweight face recognition model.

**Figure 5:**
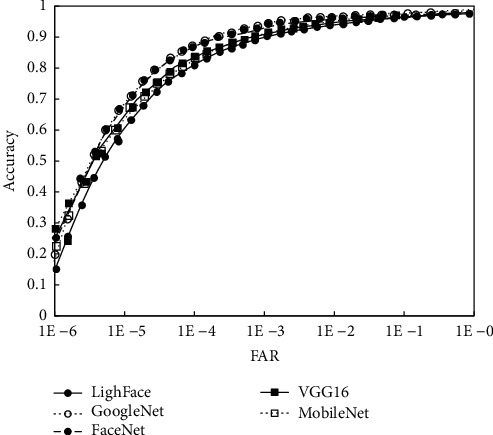
Comparison of accuracy of different models.

**Figure 6:**
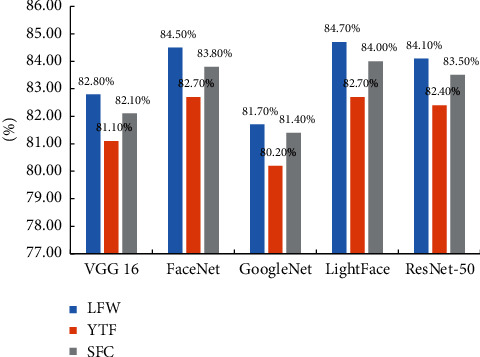
Comparison of accuracy of different models.

For the sake of further verifying the effectiveness and generalization ability of the proposed model, the research will be verified on the experimental dataset. The accuracy of different models on the LFW data is shown in Figure 5, and when FAR = 0.0001, the accuracy of different models is represented in Figure 6. It can be seen from the experimental results that compared with the comparison model, the calculation amount and parameters of the lightface model proposed in this study are greatly reduced and the accuracy is improved by 1.5%–3%. This indicates that the lightface model which is proposed in this study has certain advantages.

*(2) Robustness Test*. For the sake of verifying the practical application effect of the proposed model, this study conducted the tests on the nexus 6p mobile device, and the performance of the proposed model has been compared with the performance of classical networks such as VGG16. The results are represented in Table 3 [17]. The table shows that the lightface model which is proposed in this study meets the lightweight network standard, can be applied in mobile devices, and has certain advantages in speed and performance.

#### 4.3.3. Validation of Privacy Protection Methods

For the sake of verifying the effectiveness of the proposed privacy protection method, this study verifies it on three datasets: LFW, SFC, and YTF and compares it with DPSGD and PATE privacy protection methods [27]. The results are represented in Table 4 [17]. The table shows that compared with the comparative privacy protection methods, the privacy protection methods proposed in this study achieve the highest recognition accuracy on the three experimental datasets, and the average recognition accuracy is about 1% higher than DPSGD and 0.5% higher than PATE. This shows that the privacy protection strategy proposed in this study is effective.

According to Theorem 2, Table 5 shows the model accuracy corresponding to the differential privacy protection value (*ε*, *δ*). The table shows that compared with the model trained without noise label data, the accuracy of the model proposed in this study is reduced by 0.34%. When the failure rate is 10^−5^, a strict privacy boundary *ε*=2.05 will be generated. This shows that the model which is proposed in this study can keep a high recognition accuracy under the premise of guaranteeing privacy security [17].

## 5. Conclusion

To sum up, this study proposes a privacy protection method based on differential privacy. Through the Bayesian GAN and differential privacy algorithm, tags with privacy protection can be obtained to prevent attackers from accessing the privacy data training model directly. By using the proposed lightface lightweight face recognition model to generate labels with noise, the restored face feature vector can be gradient reduced, and the accuracy of the attacker to recover face sensitive data can be reduced. Through privacy loss analysis, an accurate privacy protection boundary can be provided for privacy data. Compared with privacy protection methods such as DPSGD and PATE, the privacy protection method proposed in this study has strong privacy protection ability and can be applied to privacy protection in APP under face recognition technology. Although this research has achieved some research results, there are still some problems in practical environment application, for example, the face recognition model based on differential privacy adopts ensemble learning in the training process, and although this method ensures that the model can maintain high accuracy, its training time is long so that the CNN network with parallel computing can be considered to speed up the training speed. In the follow-up study, from this aspect, the further discussion will be conducted.

## Figures and Tables

**Table 1:** Weight calibration module structure.

Layer	Size-in	Size-out
Avg. pool	*D*_*F*_ × *D*_*F*_ × *M*	1 × 1 × *M*
FC1	1 × 1 *M*	1 × 1 × *M*/*γ*
FC2	1 × 1 × *M*/*γ*	1 × 1 × *M*

**Table 2:** Comparison of calculation amount and parameter amount of different models.

Model	Million multiadds	Million parameters
LightFace	572	4.7
MobileNet	568	4.2
FaceNet	1600	7.5
GoogleNet	1550	6.8
VGG16	15300	138

**Table 3:** Robustness test results of different models.

Model	Time (ms)	CPU usage (%)	Power consumption (mW)	Performance per watt (1000 fps/w)	Memory usage (M)
LightFace	290.2	99.1	4438	0.937	54
SqueezeNet	125.9	98.6	4774	1.903	19
MobileNet	279.5	99	4358	0.821	52
VGG16	3418.3	99.5	4298	0.068	707

**Table 4:** Comparison of model accuracy under different methods.

Dataset	DPSGD (%)	PATE (%)	Our method (%)
LFW	97.5	98.0	98.5
YTF	95.1	95.7	96.2
SFC	96.4	97.2	97.8

**Table 5:** Comparison of model recognition accuracy under different methods.

*ε*	*δ*	Nonprivate baseline (%)	Our publish model (%)
2.05	10^−5^	99.48	99.14
4.15	10^−5^	99.48	99.14
8.13	10^−5^	99.48	99.31

## Data Availability

The experimental data are available from the corresponding author upon request.
